# Assessing root resorption incidence in clear aligner versus fixed orthodontic appliance using CBCT

**DOI:** 10.6026/973206300222933

**Published:** 2026-05-31

**Authors:** Narayana Prasad P, Tarun Sharma, Anupa Rawat, Luv Bhatt, Sumit Joshi, Sonali Bhatt

**Affiliations:** 1 Department of Orthodontics and Dentofacial Orthopedics, Seema Dental College and Hospital, Rishikesh, Uttarakhand, India

**Keywords:** Clear aligners, cone beam computed tomography(CBCT), fixed orthodontic appliance, orthodontic treatment, root resorption

## Abstract

Root resorption is a significant complication in orthodontic treatment that can lead to long-term tooth damage and affects treatment outcomes. Therefore, it is of interest to assess the incidence and severity of root resorption in patients treated with clear aligners versus fixed orthodontic appliance MBT (McLaughlin, Bennett, and Trevisi) using CBCT. Hence, a total of 100 patients (50 per group) were evaluated at baseline, 3 months and 6 months of treatment. The results showed high incidence and severity of root resorption in the fixed orthodontic appliance (MBT) group compared to the clear aligner group. Thus, data offers insights into the selection of safer treatment options.

## Background:

Root resorption is a common complication in orthodontic treatment that can affect the long-term stability and success of the therapy. It refers to the loss of tooth structure at the root surface, leading to a decrease in the root length or alterations in the root shape [1]. This phenomenon can be influenced by various factors, including the type of orthodontic appliance used, the duration of treatment, the force applied and individual patient factors such as genetic predisposition. Root resorption is often asymptomatic in the early stages but can result in significant damage if it progresses unchecked, potentially causing tooth mobility, discomfort and compromised aesthetics. As a result, understanding and preventing root resorption has become a significant concern for orthodontists [2]. Two popular orthodontic appliances that are commonly used today are clear aligners and fixed orthodontic appliance (MBT). Clear aligners, such as Invisalign, have gained popularity due to their aesthetic appeal and the convenience they offer patients, especially adults [3]. These appliances use a series of custom-made, removable trays to move teeth incrementally, exerting controlled and gradual forces. In contrast, fixed orthodontic appliance (MBT), often referred to as traditional braces, consist of brackets and wires that are permanently attached to the teeth, providing more continuous and precise force application [4]. The incidence of root resorption has been reported to vary between different orthodontic systems and much debate exists over whether clear aligners or fixed orthodontic appliance (MBT) result in a higher incidence of this complication. The mechanical forces involved in the two systems differ significantly [5]. Fixed orthodontic appliance (MBT) delivers constant, localized forces, which might result in a higher likelihood of root resorption due to the persistent pressure on the roots. On the other hand, clear aligners, with their more distributed force system, might present a different risk profile, as the forces are typically lighter and more dispersed over the surface of the tooth [6]. In recent years, Cone Beam Computed Tomography (CBCT) has become an essential tool in dentistry, providing highly detailed three dimensional (3D) imaging of the dental and skeletal structures [7]. CBCT offers superior visualization of root structures compared to traditional two dimensional (2D) radiographs, making it an invaluable tool for assessing root resorption *in vivo*. Through the use of CBCT, orthodontists can better understand the extent of root resorption, detect it earlier and monitor it throughout the course of treatment. This allows for more personalized and timely interventions to mitigate potential damage to the teeth [8]. Despite the growing body of research on root resorption, there is still a lack of consensus regarding which orthodontic appliance clear aligners or fixed orthodontic appliance (MBT) poses a greater risk for this complication [9]. Some studies suggest that fixed orthodontic appliance (MBT) may be associated with higher levels of root resorption due to their more rigid mechanics, while others argue that clear aligners, through generally gentler, might still result in significant root changes, especially with long-term use [10]. Therefore, it is of interest to report the incidence of root resorption in patients treated with clear aligners and fixed orthodontic appliance (MBT) using CBCT.

## Methodology: 

This study aimed to assess the incidence of root resorption in orthodontic patients treated with clear aligners versus fixed orthodontic appliance (MBT) using CBCT for detailed analysis. A total of 100 patients were selected for participation in the study, with 50 patients in each treatment group: clear aligners and fixed orthodontic appliance (MBT). All patients were recruited from a pool of individuals seeking orthodontic treatment at Department of Orthodontics & Dentofacial Orthopaedics, Seema Dental College & Hospital, Rishikesh, Uttarakhand, a period of six months. Inclusion criteria included patients aged 18-40 years, with moderate to mild malocclusions that required orthodontic treatment and no previous history of significant dental trauma or systemic conditions that could affect tooth development. Patients with active periodontal disease, those requiring surgical orthodontics, or those who had previously received orthodontic treatment were excluded. Upon enrollment, each participant underwent a comprehensive clinical examination to assess their dental and medical history. Baseline CBCT scans was taken for each participant prior to the initiation of treatment to establish a reference point for root morphology and any pre- existing root conditions. The study then followed two groups: Clear Aligner Group: Fifty patients were assigned to the clear aligner group, where they received treatment using Invisalign or a similar clear aligner system. These aligners were fabricated based on impressions and 3D imaging of each patient's dental arch. Treatment duration varied depending on the severity of the malocclusion, but all patients underwent treatment for a minimum of six months. The aligners were worn for 20-22 hours daily, with periodic adjustments every two weeks to ensure the desired tooth movement. Fixed Orthodontic appliance (MBT) Group: The remaining 50 patients were assigned to the fixed orthodontic appliance (MBT) group, where they received traditional metal or ceramic braces. Fixed orthodontic appliance (MBT) was bonded to the teeth using composite resin and the wires were adjusted every 4-6 weeks to guide the movement of the teeth. The treatment duration for this group was also at least six months, with similar orthodontic goals for each patient. During the treatment period, both groups received regular follow-up appointments for clinical assessments and monitoring of tooth movement. The primary focus was on the root resorption status, which was evaluated through CBCT scans. These scans were taken at three critical time points: baseline (before treatment), at the 3-month mark and at the 6-month follow-up. The CBCT imaging provided high-resolution, 3D images of the teeth and their roots, allowing for precise measurement of root length, shape and the extent of any resorption. The scans were analyzed using specialized software to quantify root resorption by comparing the root lengths and morphology from the baseline scan with those at the 3- and 6-month time points. Root resorption was classified as mild, moderate, or severe based on visual analysis and the degree of root shortening observed in the scans. Statistical analysis was conducted to compare the incidence and severity of root resorption between the two groups. Descriptive statistics summarized the distribution of resorption levels within each group and inferential statistics, such as the chi-square test and independent t-tests, were used to assess significant differences in root resorption between the clear aligner and fixed orthodontic appliance (MBT), groups. A p-value of less than 0.05 was considered statistically significant. This study was conducted in accordance with ethical guidelines and all participants provided written informed consent before participating. The research protocol was reviewed and approved by the Institutional Review Board (IRB) to ensure patient safety and adherence to ethical standards. Therefore, by utilizing CBCT imaging to assess root resorption, this study aimed to provide a comprehensive comparison of the two orthodontic treatment modalities, contributing valuable insights to the field of orthodontics and improving patient outcomes.

## Results:

A total of 100 patients participated in the study, with 50 patients in each treatment group. At the 6-month follow-up, 30% of the participants exhibited root resorption. As shown in Table 1, 24% of patients in the clear aligner group experienced root resorption, while 36% of patients in the fixed orthodontic appliance (MBT) group had resorption. In contrast, 76% of the clear aligner group and 64% of the fixed orthodontic appliance (MBT) group did not show any signs of root resorption. The severity of root resorption was categorized into mild, moderate and severe resorption. According to Table 2, in the clear aligner group, 20% of patients showed mild root resorption, 4% exhibited moderate resorption and no patients had severe resorption. In comparison, the fixed orthodontic appliance (MBT) group had 28% of patients with mild resorption, 6% with moderate resorption and 2% with severe resorption (Table 3). This indicates that a larger proportion of patients in the fixed orthodontic appliance (MBT) group experienced both moderate and severe root resorption compared to the clear aligner group.

## Discussion:

The present study found a higher incidence (36%) and severity of root resorption in the fixed orthodontic appliance (MBT) group compared to the clear aligner group (24%), with greater mean root length reduction in the fixed orthodontic appliance (MBT) group (0.8 mm versus 0.4 mm). This pattern aligns with several previous investigations, reinforcing the notion that orthodontic appliance type influences root resorption outcomes. Jyotirmay (2021) [1] reported that fixed orthodontic treatment resulted in significantly greater apical root resorption (ARR) than clear aligner therapy when assessed using CBCT (mean ARR of 1.51 mm versus 1.12 mm), concluding that fixed orthodontic appliance (MBT) are associated with more severe root resorption than clear aligners. This study's findings correspond with the current results, which also show greater resorption magnitude in fixed orthodontic appliance (MBT), suggesting that continuous forces in fixed mechanics could contribute to more root damage than intermittent forces in clear aligner therapy. Li *et al.*(2020) [12] demonstrated a significantly lower prevalence and severity of ARR in patients treated with clear aligners than those treated with fixed orthodontic appliance (MBT) (56.3% versus 82.1%; severity 0.13 mm versus 1.12 mm) when evaluated via CBCT. Their findings largely mirror our results in which fewer clear aligner patients exhibited resorption, supporting the conclusion that clear aligners may reduce the risk or extent of root resorption compared to fixed systems. Fang *et al.*(2019) [13] in a systematic review and meta-analysis reported that root resorption associated with clear aligner treatment was significantly lower than with fixed orthodontic appliance (MBT), noting a smaller standardized mean difference favoring clear aligners for lower resorption levels. Although meta-analytic data encompass studies of variable design, Fang *et al.'s* [13] conclusion supports our interpretation that fixed orthodontic mechanics tend to produce more root resorption than aligner therapy. Bespalez-Neto *et al.*(2025) [14] conducted a randomized clinical trial using advanced AI-assisted CBCT surface models to quantify external ARR in incisors following clear aligner versus fixed orthodontic appliance (MBT) therapy. Although the full results are pending publication, preliminary findings indicate measurable differences in 3D root morphology changes between the treatment modalities, suggesting that aligner therapy may influence root resorption patterns differently compared to fixed orthodontic appliance (MBT). This emerging evidence while focused on incisors with 3D surface analysis reinforces that appliance biomechanics affect root resorption differently. It supports the concept, seen in our findings, that aligner treatment may be less predisposed to extensive resorption compared to fixed braces. However, contrary evidence also exists, such as recent meta-analyses showing no statistically significant difference in clinically relevant root resorption between modalities, suggesting that the choice of appliance should also consider biomechanics, treatment objectives and patient preferences rather than resorption risk alone [15].

## Conclusion:

We show that patients treated with fixed orthodontic appliance (MBT) experienced high incidence and greater severity of root resorption compared to those treated with clear aligners. The results show the potential advantages of clear aligners in minimizing root resorption risk. Thus, the importance of selecting orthodontic treatment options based on both effectiveness and potential side effects like root damage is reported.

## Figures and Tables

**Table 1:** Incidence of root resorption at 6-month follow-up

**Group**	**Number of Patients**	**Percentage(%)**
With Resorption (Clear Aligner)	12	24%
With Resorption (Fixed Orthodontic appliance (MBT))	18	36%
No Resorption (Clear Aligner)	38	76%
No Resorption (Fixed Orthodontic appliance (MBT))	32	64%
Total	100	100%

**Table 2:** Severity of root resorption in both groups

**Group**	**Mild Resorption**	**Moderate Resorption**	**Severe Resorption**	**Total (%)**
Clear Aligner	10 (20%)	2 (4%)	0 (0%)	24%
Fixed Orthodontic appliance (MBT)	14 (28%)	3 (6%)	1 (2%)	36%
Total	28 (28%)	5 (5%)	1 (1%)	100%

**Table 3:** Comparison of root length reduction at 6 months

**Group**	**Mean Root Length Reduction (mm)**	**Standard Deviation (mm)**
Clear Aligner	0.4	0.2
Fixed Orthodontic appliance (MBT)	0.8	0.4
